# Elevated levels of serum amyloid A indicate poor prognosis in patients with esophageal squamous cell carcinoma

**DOI:** 10.1186/1471-2407-12-365

**Published:** 2012-08-23

**Authors:** Jun-Ye Wang, Yu-Zhen Zheng, Juan Yang, Yue-Hao Lin, Shu-Qin Dai, Ge Zhang, Wan-Li Liu

**Affiliations:** 1 State Key Laboratory of Oncology in Southern China, Sun Yat-sen University Cancer Center, Guangzhou, China; 2 Department of Thoracic Surgery, Sun Yat-sen University cancer center, Guangzhou, China; 3 Department of Clinical Laboratory Medicine, Guangzhou Medical University, Guangzhou, China; 4 Department of Clinical Laboratory Medicine, Sun Yat-sen University cancer center, 651 Dongfeng Road East, Guangzhou, 510060, China; 5 Department of Microbial and Biochemical Pharmacy, School of Pharmaceutical Sciences, Sun Yat-sen University, No.132 Waihuandong Road, University Town, Guangzhou, 510006, China

**Keywords:** Serum amyloid A, Esophageal squamous cell carcinoma, Prognosis, Biomark

## Abstract

**Background:**

Increase of Serum amyloid A (SAA) level has been observed in patients with a variety of cancers. The objective of this study was to determined whether SAA level could be used as a prognostic parameter in patients with esophageal squamous cell carcinoma (ESCC).

**Methods:**

SAA levels were measured by rate nephelometry immunoassay in 167 healthy controls and 167 ESCC patients prior to surgical resection. Statistical associations between clinicopathological observations and SAA levels were determined using the Mann–Whitney U test. The clinical value of SAA level as a prognostic parameter was evaluated using the Cox’s proportional hazards model.

**Results:**

SAA levels were significantly higher in patients with ESCC compared to levels in healthy controls (13.88 ± 15.19 mg/L vs. 2.26 ± 1.66 mg/L, *P* < 0.001). Elevation of SAA levels (≥ 8.0 mg/L) was observed in 54.5% (91/167) of patients with ESCC but not in healthy controls. SAA levels were associated with tumor size (*P* < 0.001), histological differentiation (*P* = 0.015), T classification (*P* < 0.001), clinical stage (*P* < 0.001), lymph node metastasis (*P* < 0.001) and distant metastasis (*P* < 0.001), but not with the age and gender of the patients or tumor location. Multivariate analysis revealed that patients with an elevated level of SAA (≥ 8.0 mg/L) had significantly lower 5-year survival rate than those with non-elevated SAA (< 8.0 mg/L, log-rank *P* < 0.0001).

**Conclusions:**

An elevated level of preoperative SAA was found to associate with tumor progression and poor survival in patients with ESCC.

## Background

Chronic inflammation has been recognized as a key factor that contributes to the development and progression of a wide range of malignancies [1]. A number of studies suggest that inflammatory status is a prognostic factor for many cancers [2-4]. Serum amyloid A (SAA), an acute phase reactant, is a high-density lipoprotein-associated lipoprotein. It is well-known as a modulator of inflammation and it plays a major role in the metabolism and transport of cholesterol [5]. Levels of SAA may rapidly increase by up to 1000-fold in response to acute inflammation, and it is a well-established indicator of inflammation in the body [6]. SAA is also used to assess low-grade chronic inflammation.

SAA has been investigated in various human malignancies as a predictor of cancer risk and as a prognostic parameter [7-9]. Interestingly, SAA has been recently identified by serum proteomic technologies as a potentially useful biomarker for human tumors, including renal cell carcinoma [10], lung cancer [11], melanoma [12], endometrial cancer [13] and nasopharyngeal cancer [14]. Elevated levels of SAA have been used as a non-invasive biomarker for prognosis of many cancers, such as breast cancer [15], lung cancer [16], melanoma [12], gastric cancer [17] and endometrial cancer [13].

A previous study in sera from patients with esophageal squamous cell carcinoma (ESCC) that used mass spectrometry and proteomic technologies found that SAA was upregulated and could be used to differentiate patients from healthy individuals [18]. ESCC, the major histopathological form of esophageal cancer, is one of the most lethal malignancies of the digestive tract and is the fourth most frequent cause of cancer deaths in China [19]. Inflammatory status has been reported to be a prognostic factor for ESCC. Elevated concentrations of serum C-reactive protein (CRP), another commonly used marker of inflammation, have been shown to be associated with disease progression and poor prognosis in patients with ESCC [20-22]. However, the relationship between SAA and ESCC progression is still unclear.

In this study, we measured the preoperative SAA levels in 167 patients with ESCC to evaluate the clinical value of SAA as a prognostic parameter in those patients.

## Methods

### Patients and sera

In this study, patients with elevated white blood cell count (>10 × 10^9^/L) were considered with inflammatory diseases and were excluded before the measurement of SAA. At last, 167 patients with primary ESCC treated between January 2005 and January 2007 in the Cancer Center of Sun Yat-Sen University were enrolled and considered as the case group. In the cohort, 129 patients were male and 38 patients were female. There were 2 cases with atherosclerotic disease and 6 cases with COPD in group with serum SAA levels≧8.0 mg/L; there were 3 cases with atherosclerotic disease and 4 cases with COPD in group with serum SAA levels < 8.0 mg/L. The patients ranged in age from 38 to 81 years (mean, 58.5 years); none had received radiotherapy or chemotherapy prior to surgery. The patient characteristics are described in Table 1. Surgical resection in this cohort of patients consisted of transthoracic en bloc esophagectomy with two- or three-field lymphadenectomy. All of the patients were classified according to the TNM classification system and resected specimens underwent pathological examination [23]. A total of 123 patients with ESCC underwent surgical resection only, 44 patients received additional platinum-based adjuvant chemotherapy and/or radiotherapy at the discretion of the surgeon and/or oncologist.

**Table 1:** Relationship between the SAA concentration and the clinicopathological variables in 167 patients with esophageal carcinoma

**Variables**	**Cases**	**SAA(mg/L)**	**Significance**	**SAA**	**SAA**	**Significance**
	**(n)**	**(Mean±SD)**	(*P*)*	**<8.0 mg/L**	**≥8.0 mg/L**	(*P*)*
Gender						
Male	129	14.89±15.72	0.084	54	75	0.126
Female	38	10.62±13.01		21	17	
Age (y)						
<60	83	12.57±13.58	0.083	43	40	0.086
≥60	84	15.41±16.74		32	52	
Location						
Up	49	15.32±17.36	0.381	23	26	0.361
Middle	88	12.61±14.51		43	45	
Low	30	15.80±13.81		10	20	
Tumor diameter						
<50 mm	102	8.02±9.30	<0.001	66	36	<0.001
≥50 mm	65	23.15±17.93		10	55	
Histological differentiation						
Well	53	5.91±3.59	0.015	35	18	<0.001
Moderate	73	16.76±8.88		27	46	
Poor	21	17.42±17.22		4	17	
pT classification						
T1	11	5.06±4.79	<0.001	7	4	<0.001
T2	23	7.17±6.81		16	7	
T3	93	12.96±13.59		42	51	
T4	40	22.08±20.02		6	34	
pN classification						
No	72	9.99±12.88	0.006	43	29	<0.001
Yes	95	17.03±16.38		29	66	
pMetastasis						
No	132	10.21±10.97	<0.001	68	64	<0.001
Yes	35	27.92±20.42		4	31	
Stage						
I	17	5.82±4.81	<0.001	13	4	<0.001
IIa	25	6.90±13.76		16	9	
IIb	25	8.82±7.88		15	10	
III	61	11.68±9.60		26	35	
IV	39	27.73±20.58		3	36	

Overall survival (OS) was defined as the interval between the date of surgery and the date of death or the date of the last known follow-up visit. Two patients died of causes unrelated to ESCC. The follow-up data from the ESCC patients in this study were available and complete.

Sera from 167 healthy volunteers (129 males, 38 females) with ages ranging from 40 to 70 years (mean: 57.8 years) were collected in physical examination, there were 1 case with atherosclerotic disease and 2 cases with COPD. Healthy controls were selected from an archive of blood samples; the control samples were matched as closely as possible to the ESCC group for sex, previous handling and the time period of sample collection.

A 5-ml blood sample from each participant (ESCC patients and health controls) was allowed to clot for 30 to 60 min at room temperature. Each clotted samples was centrifuged at 1,500 g for 10 min. All sera were aliquoted and frozen at −70°C until use. This study was approved by the Institute Research Ethics Committee of the Cancer Center of Sun Yat-Sen University and informed consents were obtained from all participants before theirs sera were used.

### Immunoassay of SAA

The concentration of SAA was determined in both the ESCC sera and the healthy control sera using rate nephelometry performed on a BN ProSpec System (Siemens, Germany) with an SAA assay kit based on a polyclonal antibody (Siemens Healthcare Diagnostics Products GmbH, Germany). The normal level for SAA in the healthy Chinese population is < 8.0 mg/L according to the kit's protocol.

### Statistical analysis

All statistical analyses were carried out using the SPSS 16.0 statistical software package (SPSS Inc., Chicago, IL). The Mann–Whitney U test was used to evaluate the difference in SAA concentrations between ESCC patients and healthy controls and to analyze the association between SAA levels and the observed clinicopathological characteristics of patients with ESCC. Pearson's chi-squared test was used to analyze the relationship between SAA levels and gender. Survival curves were plotted by the Kaplan-Meier method and compared using the log rank test. The significance of various variables for survival was analyzed using the Cox proportional hazards model (univariate and multivariate analysis). *P* < 0.05 was considered to be statistically significant in all cases.

## Results

**Figure 1:**
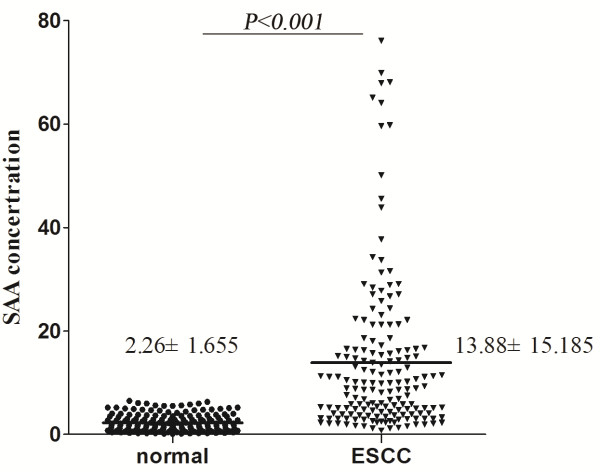
**Scatter plots of preoperative serum SAA levels in patients with ESCC (n = 167) and in healthy controls (n = 167).** There was a significant difference between these two groups (*P* < 0.001). Abnormally high levels of SAA were found only in the patient group.

Figure 1 shows the level of SAA in the healthy controls (n = 167) and ESCC patients (n = 167), respectively. The mean SAA levels in the healthy controls and ESCC patients prior to surgery were 2.26 mg/L (SD, 1.66; range, 0.10-6.50) and 13.88 mg/L (SD, 15.19; range, 0.76-76.1), respectively. SAA values from ESCC patients were significantly higher than those from the healthy controls (*P* < 0.001).

Extremely high levels of SAA were found only in the patient group. The maximum concentration of SAA that was regarded as normal was set at 8.0 mg/L, as specified by the SAA assay kit. Elevation of the SAA levels at or above this value (≥ 8.0 mg/L) were observed in 54.5% (91/167) of patients with ESCC, however, none of the 167 healthy controls had serum SAA levels above 8.0 mg/L.

The associations between median serum SAA levels and clinicopathological parameters are presented in Table 1. SAA levels were not associated with age, gender and tumor location; however, elevated meadian SAA levels were significant for patients with larger tumors (≥ 50 mm; *P* < 0.001) and for those with more advanced disease, including lymph node metastasis (*P* = 0.006) and distant metastasis (*P* < 0.001). There was also an association between raised SAA levels and poorly differentiated tumors (*P* = 0.015). We also noted that the median levels of SAA significantly increased with increasing T classification (*P* < 0.001) and clinical stage (*P* < 0.001) of the malignancy.

Next, patients were classified into two groups according to their SAA level (< 8.0 mg/L vs. ≥ 8.0 mg/L); the relationships between SAA levels and clinicopathological parameters were assessed. No significant difference in age, gender and tumor location was found between the two groups; however, patients with poor histological differentiation (*P* < 0.001), larger tumor size (*P* < 0.001), higher T classification (*P* < 0.001), lymph node metastasis (*P* < 0.001), distant metastasis (*P* < 0.001) and higher clinical stage (*P* < 0.001) were more frequently observed in the elevated SAA group (SAA ≥ 8.0 mg/L) than in the non-elevated SAA group (SAA < 8.0 mg/L).

**Figure 2:**
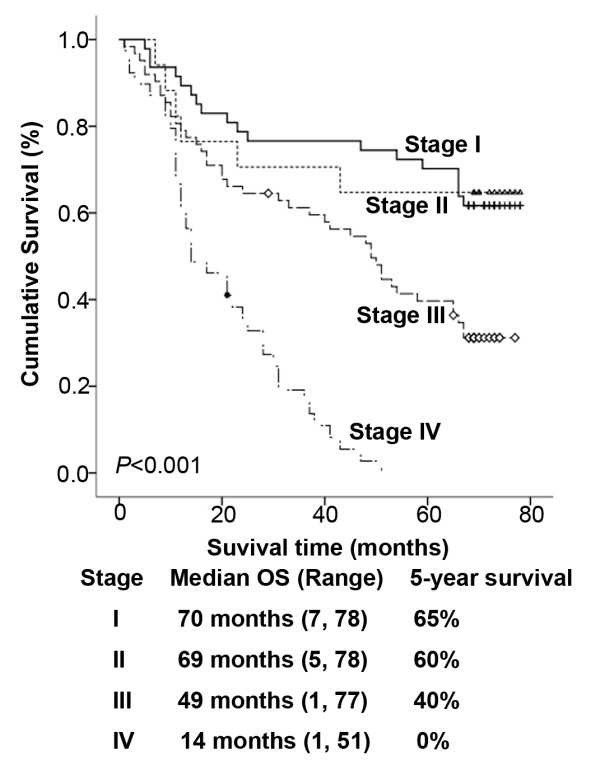
Comparison of overall survival between different stages.

The overall survival of patients with ESCC was plotted using the Kaplan-Meier method and a log-rank test was employed to evaluate the prognostic significance of SAA levels. Of the entire cohort, the predicted five-year overall survival rate was 41%, with a median survival time of 45.0 months (range from 1 month to 78 months). Figure 2 shows the comparison of survival time between different stages.

**Figure 3:**
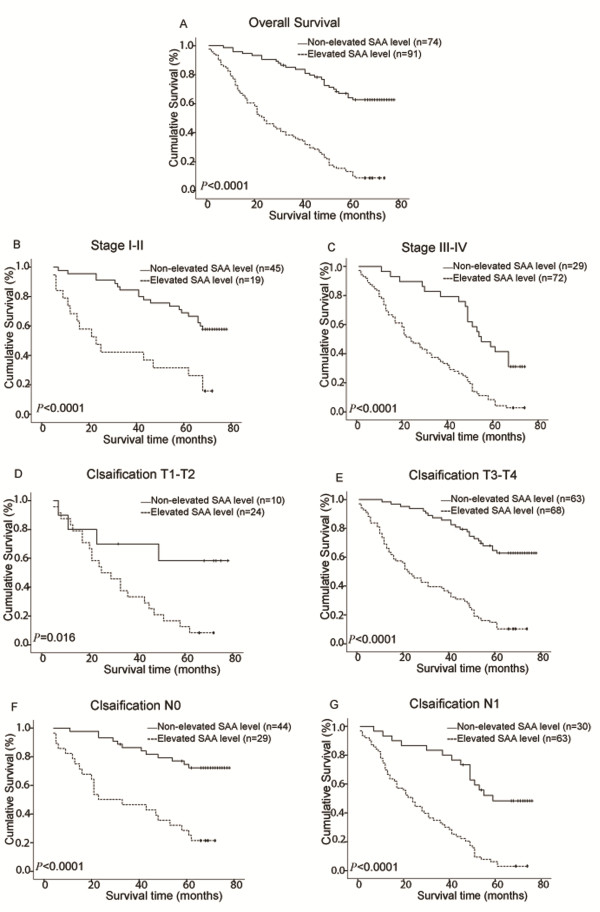
**Overall survival curves for patients with ESCC after curative resection.** The patients were categorized with elevated (≥ 8.0 mg/L) or non-elevated (< 8.0 mg/L) levels of SAA. The *P*-values were determined using the log rank test. all patients (**a**); clinical stage I-II subgroup (**b**); clinical stage III-IV subgroup (**c**); T1-T2 subgroup (**d**); T3-T4 subgroup (**e**); N0 subgroup (**f**); N1 subgroup (**g**.)

The group with non-elevated SAA levels (< 8.0 mg/L) showed a significantly better 5-year survival rate than the elevated SAA group (≥ 8.0 mg/L; Figure 3a). The cumulative 5-year survival rate in the non-elevated SAA group was 64.1%, whereas it was only 13.2% in the elevated SAA group (log-rank *P* < 0.0001).

We also analyzed the prognostic value of SAA levels in selective patient subgroups stratified according to the disease stage, and T and N classifications, respectively. ESCC patients with elevated SAA levels had significantly shorter overall survival rate compared to patients with non-elevated SAA levels in the clinical stage I-II subgroup (n = 64; log-rank *P* < 0.0001; Figure 3b), the clinical stage III-IV subgroup (n = 101; log-rank *P* < 0.0001; Figure 3c), the T1-T2 subgroup (n = 34; log rank *P* = 0.016; Figure 3d), the T3-T4 subgroup (n = 131; log rank, *P* < 0.0001; Figure 3e), the N0 subgroup (n = 72; log rank, *P* < 0.0001; Figure 3f) and the N1 subgroup (n = 93; log-rank, *P* < 0.0001; Figure 3g).

Results of univariate Kaplan-Meier analysis and the multivariate Cox regression model with respect to overall survival rate are shown in Table 2. Clinical stage, N classification and SAA levels are significantly correlated with survival in univariate Kaplan-Meier analysis (log rank test *P* < 0.001) respectively. To determine whether SAA levels could be used as an independent prognostic factor for outcomes we performed a multivariate analysis for survival, based on SAA levels, age, clinical stage, N classification and gender. In this analysis, clinical stage (log rank test *P* = 0.047), N classification (log rank test *P* < 0.001) and SAA levels (log rank test *P* < 0.001) were recognized as independent prognostic factors (Table 2). Thus, our findings indicate that the preoperative serum SAA level is an independent prognostic factor for esophageal squamous cell carcinoma.

**Table 2:** Univariate and multivariate survival analysis in patients with esophageal carcinoma

**Characteristic**	**Univariate analysis**			**Multivariate analysis**		
	**HR**	**95% CI**	*P*value*	**HR**	**95% CI**	*P*value*
Age, years						
< 60 vs. ≥60	1.242	0.847-1.823	0.267	1.254	0.835-1.789	0.302
Gender						
Male vs. Female	0.822	0.510-1.327	0.423	0.568	0.316-0.863	0.093
pN metastasis						
Yes vs. No	1.923	1.112-2.545	<0.001	1.897	1.237-2.675	0.001
pTNM stage						
I-II vs. III-IV	3.3500	2.112-5.315	<0.001	1.636	1.005-2.661	0.047
SAA level						
<8.0 mg/L vs.	12.217	7.162-20.842	<0.001	6.762	2.941-15.548	<0.001
≥8.0 mg/L						

Abbreviations: HR, Hazard ratio; 95% CI, 95% confidence interval; * Cox hazard regression modal.

## Discussion

Inflammation appears to play an important role in esophageal carcinogenesis [24]. Mechanisms of inflammation-associated tumor development have been well studied [25]. Key players in the inflammatory cascade include cytokines and inflammatory enzymes.

SAA, a well-established indicator of inflammation, is a nonspecific, acute-phase, hepatic protein secreted in response to cytokines such as interleukin-1, interleukin-6 and tumor necrosis factor α [26]. SAA participates in cholesterol transport, extracellular matrix degradation and the recruitment of inflammatory cells [5]. Accumulating evidence has suggested that SAA might be used in the clinic to detect a pattern of physiological events that could indicate the growth of a malignancy and/or a host response [7]. Elevated SAA levels have been observed in patients with renal cancer [10], melanoma [12], endometrial cancer [13], nasopharyngeal carcinoma [14], lung cancer [16], gastric cancer [17], colorectal cancer [27] and breast cancer [15]. A previous study using mass spectroscopy found that serum SAA levels increased significantly in postoperative ESCC patients compared with preoperative patients and healthy subjects [18]. However, this study did not determine whether the elevated levels of SAA were related to ESCC or the trauma of surgery.

In this study, we first showed that about 6.1-fold higher levels of SAA in ESCC patients' preoperative sera compared to healthy controls (13.88 mg/L ± 15.19 mg/L vs. 2.26 mg/L ± 1.66 mg/L). We have demonstrated that the preoperative serum SAA level may be an independent and important prognostic indicator in patients with ESCC following curative esophageal resection. The SAA concentration correlated significantly with unfavorable clinicopathological factors such as tumor size, T stage, clinical stage, lymph node metastasis and distant metastasis.

In present study, we have obtained similar prognosis compared with previous studies [28,29]. However, the survival curve was indiscriminate between stage I and II (*P* = 0.976), this may be contributed to the small sample size and, most patients of stage II were nodal negative.

The overall survival rate among patients with elevated SAA (≥ 8.0 mg/L) was significantly lower compared with the rate in the group of patients with non-elevated SAA levels (< 8.0 mg/L). Patients with elevated SAA levels had shorter survival durations compared to those with non-elevated SAA levels. The current data are consistent with previous studies that support the use of SAA as an independent prognostic marker in renal cancer [10], melanoma [12] and lung cancer [16].

It has been reported that SAA is produced by the liver and enters the systemic circulation in response to stimulation by inflammatory cytokines, such as interleukin-6 (IL-6). IL-6 is a potent proinflammatory cytokine and is produced at local tissue sites. It stimulates the liver to produce acute phase proteins, including SAA [26]. Elevated serum levels of IL-6 have been shown to be correlated with disease progression and poor prognosis in patients with esophageal cancer [30]. It has also been observed that IL-6 expression is elevated (increases in both mRNA and protein concentrations) in esophageal tumor tissues; serum levels of IL-6 have also been correlated with tumor volume. In present study, our data show that gradual elevation of serum SAA levels are associated with increasing T classification, increasing clinical stage as well as disease progression. Based upon these results, we speculate that the rise in SAA may be caused by excess inflammatory cytokines, in particular IL-6, which has previously been shown to be produced by the large number of cancer cells in patients with advanced esophageal carcinoma [31].

On the other hand, elevated SAA levels may also be a primary product of tumors. Highly expressed SAA has been observed in lung cancer tissues and in vitro experiments have shown that the SAA protein can be induced in lung cancer cells by their interaction with THP-1 monocytes [11]. The secretion of SAA by tumor cells has also been measured in renal cancer [10], endometrial carcinoma [13], colorectal cancer [27] and ovarian carcinoma [32]. However, it remains unknown whether esophageal tumor cells express SAA; further investigations are needed to determine whether ESCC tumor tissue-derived cytokines stimulate SAA synthesis in esophageal epithelial cells and thus contribute to elevated circulating SAA levels.

The functions of the SAA protein, described in the context as inflammation, are compatible with the mechanism of tumor invasion and metastasis. A previous study in animal models has demonstrated that the overexpression of SAA promoted Lewis lung carcinoma (LLC) cells to metastasize and colonize in the lung [11]. It was found that SAA bound to extracellular matrix components with results of subsequent potential modification of cell binding, enhancement of plasminogen activation, stimulation of matrix metalloproteinase (MMP) production, and increase of the invasive potential of tumor cells [33]. It was also reported that SAA stimulated M-CSF and MCP-1 expression in hepatocellular carcinoma cells; and these factors skewed M1 tumor-associated macrophages into M2 tumor-associated macrophages, which exacerbated HCC invasion both in vitro and in vivo [34]. Additionally, SAA-1 could induce anti-inflammatory interleukin-10 (IL-10)-secreting neutrophils, which have been linked to the consequent promotion of melanoma progression [35]. All these known effects of SAA in tumor invasion and metastasis may explain our findings that significantly higher concentrations of SAA were observed in patients with lymph node or distant metastatic disease, and patients with an elevated level of SAA (≥ 8.0 mg/L) had significantly lower 5-year survival rate than those with non-elevated SAA (< 8.0 mg/L).

## Conclusion

In conclusion, our study revealed that an elevated level of preoperative serum SAA was significantly associated with progressive disease and reduced survival durations in patients with ESCC. This finding might serve as the basis to explain the poor progression of patients who have undergone ESCC resection. The measurement of SAA is simple, cheap and well-established in clinical chemistry laboratories. This immunoassay could provide a relatively inexpensive and objective clinical prognostic tool for the assessment of ESCC progression.

## Abbreviations

SAA: serum amyloid A; ESCC: esophageal squamous cell carcinoma; CRP: C-reactive protein; OS: overall survival; SD: standard deviation; IL: interleukin; LLC: Lewis lung carcinoma; COPD: chronic obstructive pulmonary disease; MMP: matric metalloproteinase; M-CSF: macrophage colony stimulating factor; MCP: monocyte chemoattractant protein; HCC: hepatocellular carcinoma.

## Competing interests

The authors declare that they have no competing interests.

## Authors’ contributions

J-YW drafted the manuscript. Y-ZZ and JY performed statistical analysis. GZ and W-LL participated in the study design and coordination. S-QD collected serum specimen. Y-HL performed the follow-up. All the authors have read and approved the final manuscript.

## Pre-publication history

The pre-publication history for this paper can be accessed here:

http://www.biomedcentral.com/1471-2407/12/365/prepub
